# Japanese Encephalitis Virus Vaccination Elicits Cross-Reactive HLA-Class I-Restricted CD8 T Cell Response Against Zika Virus Infection

**DOI:** 10.3389/fimmu.2020.577546

**Published:** 2020-09-25

**Authors:** Marion Tarbe, Wei Dong, Guang Hu, Yongfen Xu, Jing Sun, Solene Grayo, Xianyang Chen, Chengfeng Qin, Jincun Zhao, Li Liu, Xiuzhen Li, Qibin Leng

**Affiliations:** ^1^The Joint Center for Infection and Immunity, Guangzhou Institute of Pediatrics, Guangzhou Women and Children’s Medical Center, Guangzhou Medical University, Guangzhou, China; ^2^Institut Pasteur of Shanghai, University of Chinese Academy of Sciences, Chinese Academy of Sciences, Shanghai, China; ^3^Affiliated Cancer Hospital & Institute of Guangzhou Medical University, State Key Laboratory of Respiratory Disease, Guangzhou, China; ^4^State Key Laboratory of Respiratory Disease, Guangzhou Institute of Respiratory Health, The First Affiliated Hospital of Guangzhou Medical University, Guangzhou, China; ^5^Department of Virology, State Key Laboratory of Pathogens and Biosecurity, Beijing Institute of Microbiology and Epidemiology, Beijing, China

**Keywords:** JEV, Zika, cross-reactive, epitope, CD8 T cells, HLA-A2 transgenic mice, heterologous immunity

## Abstract

Japanese encephalitis virus (JEV) exposure or vaccination could elicit cross-reactive CD8 T cell immunity against heterologous flaviviruses in humans. In addition, cross-reactive CD8 T cells induced by dengue virus (DENV) have been shown to play a protective role against Zika virus (ZIKV). However, how JEV exposure or vaccination affects ZIKV infection in humans remains unclear. In this report, epitope prediction algorithms were used to predict the cross-reactive CD8 T cell epitope restricted to human HLA between JEV and ZIKV. We found that these predicted CD8 T cell epitopes are immunogenic and cross-reactive in humanized HLA transgenic mice. Moreover, JEV vaccine immunization provided cross-protection against ZIKV infection. Furthermore, CD8 T cells were involved in the protection against ZKIV infection *in vivo*. Our results have an important clinical implication that vaccination with JEV SA14-14-2 may provide protection against ZIKV infection in humans.

## Introduction

Zika virus (ZIKV) is a global health threat due to its association with severe congenital malformations and its widespread transmission (1, 2). Nevertheless, the spreading of ZIKV is limited in China and South-East Asia (3–5) despite the presence of ZIKV transmission-competent mosquitoes and the circulation of other flaviviruses, including dengue viruses (DVs) and Japanese encephalitis virus (JEV). Several hypotheses have been suggested to explain the low incidence of ZIKV infection in Asia: low burden of public health prior to the Micronesia epidemic in 2007, misdiagnosis even in the laboratory due to the presence of other flaviviruses, endemic of ZIKV in Asia for several decades potentially providing long-lasting immunity, and/or cross-protective immunity provided by other endemic flaviviruses (6). While cross-reactivity of DV immunity with ZIKV infection has been reported because of the presence of DV in South America (7–9), little is known about immunologic cross-reactivity of JEV to ZIKV and whether JEV pre-existing immunity may provide protection or contribute to ZIKV pathogenesis.

JEV is an arthropod-borne virus transmitted mainly through the bite of Culex species mosquitoes, primarily *Culex tritaeniorhynchus*. JEV is endemic to South and South-East Asia and epidemic in North Asia. It is reported that JEV seropositivity in adults in Korea range from 79–94% (10). Thus, JEV circulates from Pakistan to Japan and from Korea to Indonesia, as well as through east Pacific regions and northern Australia (11). JEV infection induces disease mainly during childhood. Approximately 10% of infected children develop mild febrile illness, and 0.1 to 1% of them progress to encephalitis, of which 20–30% are fatal and 30–50% result in permanent neurologic sequelae (12). The remainder of JEV infections in humans are clinically silent. The overwhelming majority of JEV-exposed individuals thus develop long-lasting immunity.

Live attenuated JEV vaccine (SA14-14-2) is widely used in China and other Asian countries (13). This vaccine is able to elicit both humoral and cellular immunity. Interestingly, while JEV vaccination in children provides high and long-lasting protection through neutralizing antibodies (14, 15). The vaccination in adults provides a T cell immune response that is more potent than a humoral response (16). Additionally, CD8 T cells in JEV-vaccinated and JEV-exposed healthy human subjects cross-react with DVs (16, 17) and other flaviviruses. Furthermore, Recent studies have revealed that murine MHC I-restricted CD8 T cells from mice infected with JEV or vaccine strain are indeed cross-reactive with ZIKV (18, 19). These evidences suggest JEV vaccination may elicit cross-reactive T cell immunity against ZIKV and thereby affect ZIKV pathogenesis in humans. To test this hypothesis, we performed bioinformatic analysis to predict the cross-reactive epitope between JEV and ZIKV. Then, the immunogenicity of these epitopes and the cross-protective role of CD8 T cells against ZIKV infection were determined in HLA-transgenic mice. Our results imply that JEV vaccination has a potential to protect ZIKV infection in humans by cross-reactive CD8 T cell immunity against these epitopes.

## Results

### Prediction of Potential Cross-Reactive Epitope to ZIKV Restricted to Human MHCs

**FIGURE 1:**
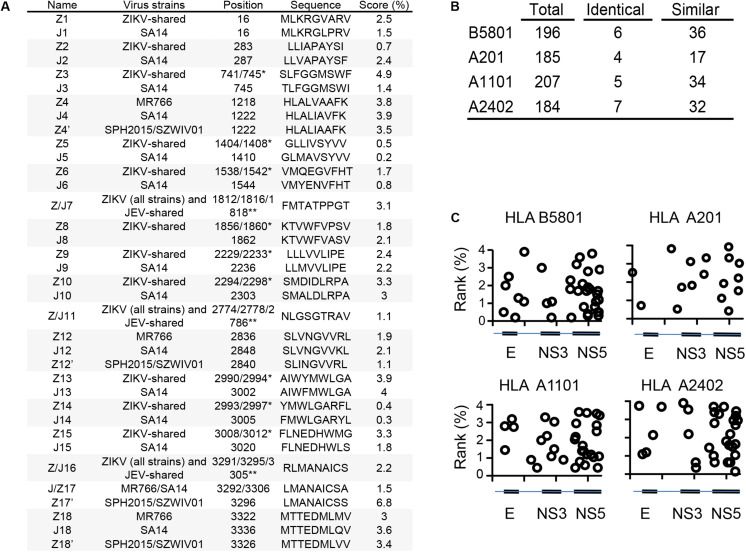
Prediction of JEV and ZIKV-derived cross-reactive HLA-B*58:01, HLA-A*02:01, HLA-A*11:01 and HLA-A*24:02-restricted epitope candidates. **(A)** Sequence of predicted HLA-A*02:01-restricted JEV SA14, ZIKV MR766, ZIKVSPH2015 and ZIKVSZWIV01 cross-reactive epitopes. **(B)** Number of predicted JEV-derived HLA-B*58:01, HLA-A*02:01, HLA-A*11:01 and HLA-A*24:02-restricted epitopes and their comparison with ZIKV MR766-derived epitopes. **(C)** Location of predicted cross-reactive epitopes on the JEV polyprotein sequence and IEDB percentage ranks. *Starting position in MR766 (-/) and SPH2015/SZWIV01 (/-); **Starting position in MR766 (-/), SPH2015/SZWIV01 (/-/) and SA14 (/-).

Human leukocyte antigen (HLA)-B^∗^58:01(B58), HLA-A^∗^02:01 (A2), HLA-A^∗^11:01 (A11) and HLA-A^∗^24:02 (A24) are prevalent MHC-I molecules in the Asian population. Moreover, HLA-A^∗^02:01 is the most prevalent MHC-I in the Caucasian population. As JEV SA14-14-2 and ZIKV polyproteins share 56% sequence homology, we wondered whether JEV vaccination could induce cross-reactive CD8 T cell response against ZIKV infection in humans. To determine the potential JEV/ZIKV cross-reactive epitope restricted to human MHC-I, we predicted antigenic peptides from the JEV SA14-14-2 polyprotein using Immune Epitope Database Analysis Resource (IEDAR) software and selected candidates presenting IEDAR scores under 4, thereby obtaining 196, 185, 207, and 184 epitopes restricted to HLA B58, HLA A2, HLA A11, and HLA A24, respectively. We then aligned the sequences of the JEV SA14-14-2 polyprotein with ZIKV MR766 (reference strain) and ZIKV SPH2015 (Brazilian strain) to determine identical or similar (no more than 2 mutations) antigenic peptides in BLAST software. Further analysis with IEDAR revealed that 42 HLA B58, 21 HLA A2, 39 HLA A11 and 39 HLA A24-binding candidates were predicted, respectively (Figure 1A and Supplementary Tables 1–3). Among them, 6 HLA B58, 4 HLA A2, 5 HLA A11 and 7 HLA A24-binding candidates were identical between SA14-14-2 and ZIKV (Figure 1B). Moreover, most of these cross-reactive candidates were located in E, NS3 and NS5 proteins of ZIKV (Figure 1C). These data imply that JEV vaccination may elicit a cross-reactive CD8 T cell response to ZIKV in humans.

### A Dominant JEV Epitope Elicits Cross-Reactive CD8 T Cell Response to ZIKV Infection in HLA A2-Transgenic Mice

**FIGURE 2:**
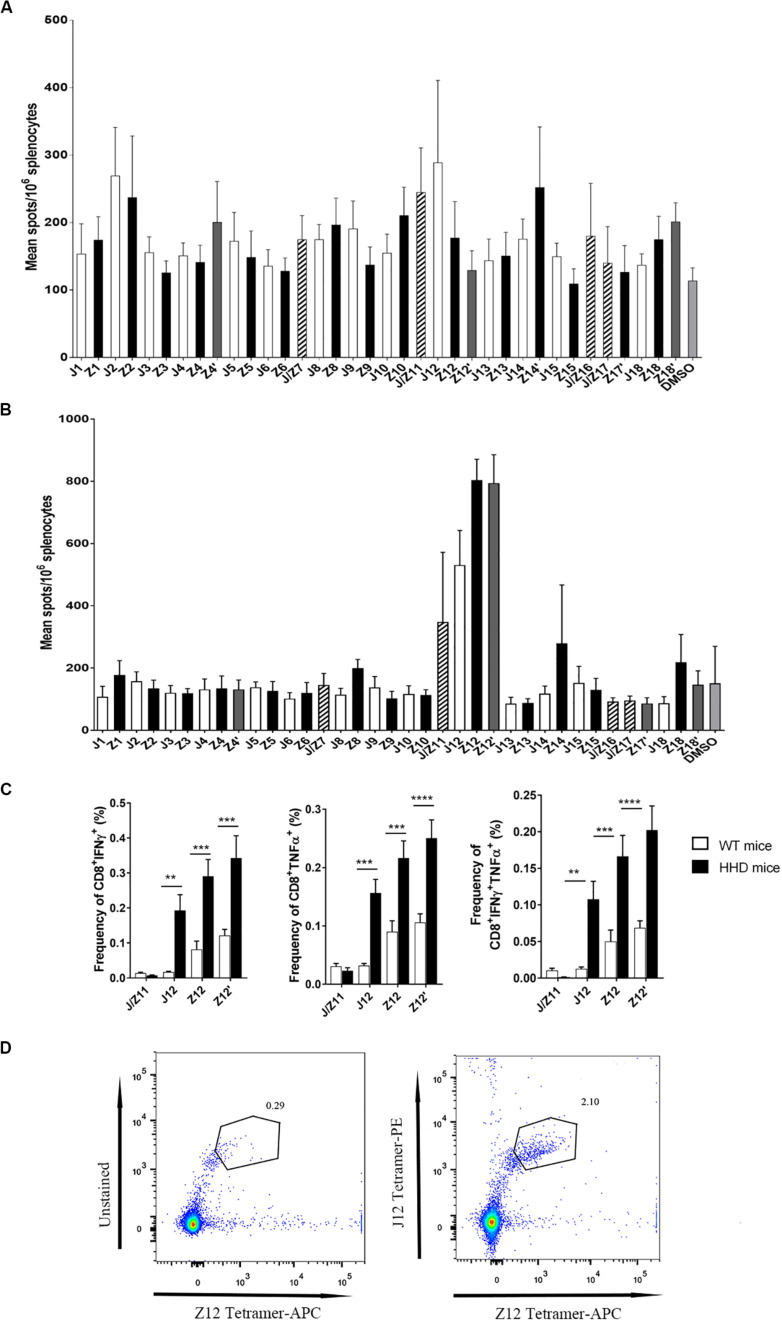
Identification of JEV and ZIKV epitopes recognized by JEV- and ZIKV-specific CD8^+^ T cells in HHD mice. **(A)** IFNγ ELISpot assay of splenocytes from JEV SA14-14-2-immunized HHD mice (*n* = 5) stimulated with the indicated peptides. **(B)** IFNγ ELISpot assay of splenocytes from ZIKV SZWIV01-infected HDD mice stimulated with the indicated peptides. **(C)** Intracellular staining of splenocytes from ZIKV SZWIV01-infected HDD mice. Splenocytes were stimulated with J/Z11, J12, Z12 and Z12’ peptides. ZIKV SZWIV01-infected WT mice served as negative controls. Cells were gated on both CD8 and TCRβ positive. **(D)** HLA A2-J12 and Z12 tetramer staining of splenocytes from ZIKV SZWIV01-infected HDD mice.

Considering the availability of HLA transgenic mice and HLA genotype prevalence in humans, we focused our study on the HLA A2-restricted epitopes and evaluated the immunogenic properties of these potential cross-reactive epitopes *in vivo*. HHD mice, which are transgenic mice with a C57B/L6 background that express the human MHC-I A2 epitope-binding site (20), were infected either with the JEV-attenuated strain SA14-14-2 or ZIKV SZ-WIV01. Seven days after infection, splenocytes were collected and stimulated with each HLA A2-restricted peptide in an IFNγ-ELISpot assay. As shown in Figure 2A, J2, Z2, J/Z11, J12, Z12, Z12’ and Z14’ peptides were immunogenic epitopes in JEV infection. However, in ZIKV infection, the T cell response to J2 and Z2 peptides was negligible. J12, Z12 and Z12’ peptides are the most dominant epitopes in response to ZIKV infection (Figure 2B). An intracellular staining assay confirmed that CD8 T cells from ZIKV-infected HHD mice indeed responded to all J12, Z12 and Z12’ peptides by producing IFNγ. Moreover, CD8 T cells that responded to J12, Z12 and Z12’ peptides were polyfunctional because they produced both IFNγ and TNFα (Figure 2C).

To further determine the cross-reactivity between J12 and Z12-specific CD8 T cells, J12 and Z12 tetramer were used to stain CD8 T cells collected from ZIKV-infected mice. We found that Z12 and J12 dual -reactive cells are significantly higher than Z12-specific CD8 T cells (Figure 2D). These results indicate that Z12-specific CD8 T cells are cross-reactive with J12-specific CD8 T cells. Altogether, these results demonstrate that JEV vaccination induces cross-reactive HLA A2-restricted CD8 T cells to ZIKV.

### JEV Vaccination Polarizes CD8 T Cell Response to the Cross-Reactive ZIKV Epitope

**FIGURE 3:**
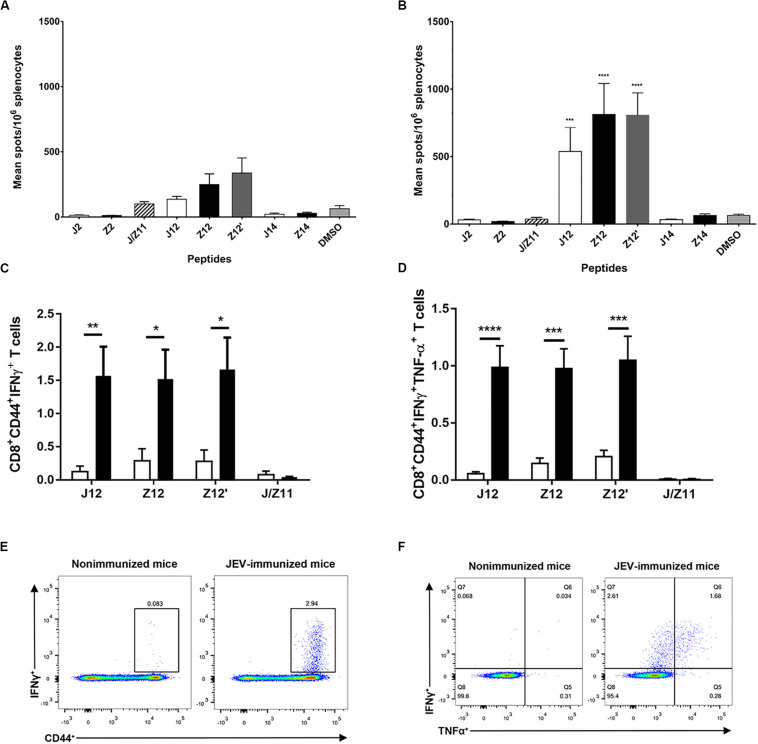
JEV-specific CD8 T cells are recalled upon ZIKV infection in HHD mice. **(A,B)** IFNγ ELISpot assay of indicated epitopes with splenocytes from HHD mice (*n* = 5 each) that were immunized without **(A)** or with **(B)** the JEV SA14-14-2 vaccine once and then infected with ZIKV. **(C,D)** Intracellular staining of splenocytes from HHD mice (*n* = 5 each) that were immunized with or without the JEV SA14-14-2 vaccine once and then infected with ZIKV. The splenocytes were cultured with indicated peptides before intracellular staining. The averages of IFNγ^+^ **(C)** and IFNγ^+^TNFα^+^ **(D)** gated on CD8 T cells. **(E,F)** Representative plot of IFNγ and TNFα expression in CD8 T cells.

We next studied whether JEV vaccination could affect the CD8 T cell response to ZIKV infection *in vivo*. HHD mice were first immunized with the JEV SA14-14-2 vaccine and then infected with ZIKV 28 days later. Non-immunized HHD mice that received ZIKV infection were served as control group. Five days post ZIKV infection, splenocytes were harvested. ELISpot was used to determine the CD8 T cell response to ZIKV epitopes. As expected, T cells responded to J12, Z12, and Z12’ peptides in Non-immunized group because of ZIKV infection. Notably, the responses to these peptides were 2 to 4 times higher in the JEV-immunized group than in the non-immunized group (Figure 3A).

We next used ICS assay to examine the IFNγ and TNFα production of CD8 T cells from JEV-immunized mice in response to peptide J12, Z12, and Z12’. As shown in the Figure 3B, the proportions of IFNγ producing CD8 T cells in the JEV-immunized group in response to J12, Z12 and Z12’ peptides were approximately 4 times higher than that in the non-immunized group. Furthermore, IFNγ-producing T cells appeared polyfunctional, as nearly 50% of them also expressed TNFα (Figures 3C,D). In addition, the majority of IFNγ and TNFα-producing T cells expressed high levels of CD44 molecules (Figures 3E,F), a memory phenotype, indicating the T cell responses are a recall response. These data suggest that JEV vaccination polarizes CD8 T cell response to a dominant epitope that cross-reacts with ZIKV.

### JEV-Specific CD8 T-Cells Provide Cross-Protection Against ZIKV Infection

**FIGURE 4:**
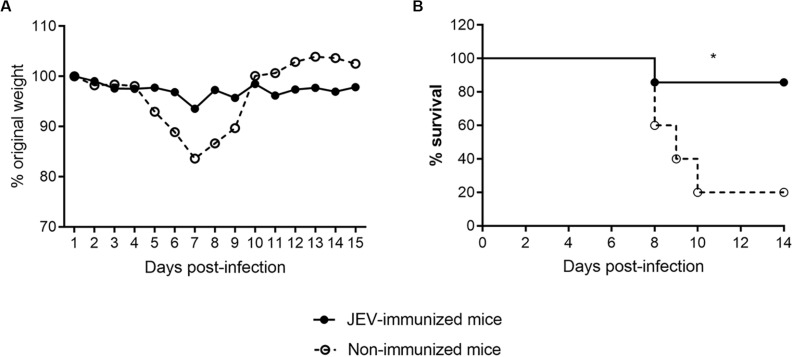
JEV immunization provides protection against ZIKV infection in HHD *Ifnar1*^–/–^ mice **(A,B)**. Body weight **(A)** and survival rate **(B)** of HHD *Ifnar1*^–/–^ mice (*n* = 5 each) that were injected with JEV SA14-14-2 vaccine (*n* = 7) or mock (*n* = 5) two times on days 0 and 28 and then infected with ZIKV on day 14 after the boost.

Next, the protective potential of the JEV cross-reactive CD8 T cells against ZIKV infection was determined *in vivo*. We first generated HHD *Ifnar1*^–/–^ mice that are susceptible to ZIKV by crossing HHD mice with *Ifnar1*^–/–^ mice. HHD *Ifnar1*^–/–^ mice were immunized twice with JEV on days 1 and 28, and then mice were challenged with ZIKV 14 days after the second immunization. The non-immunized mice lost approximately 17% of their body weight from day 4 to day 7 after ZIKV infection (Figure 4A). Eighty percent of these mice died 10 days after ZIKV infection (Figure 4B). In contrast, the immunized mice lost little body weight, and only 1 out of 7 mice died after ZIKV infection (Figures 4A,B). These observations suggest that JEV vaccination protects humanized HHD transgenic mice from ZIKV infection.

**FIGURE 5:**
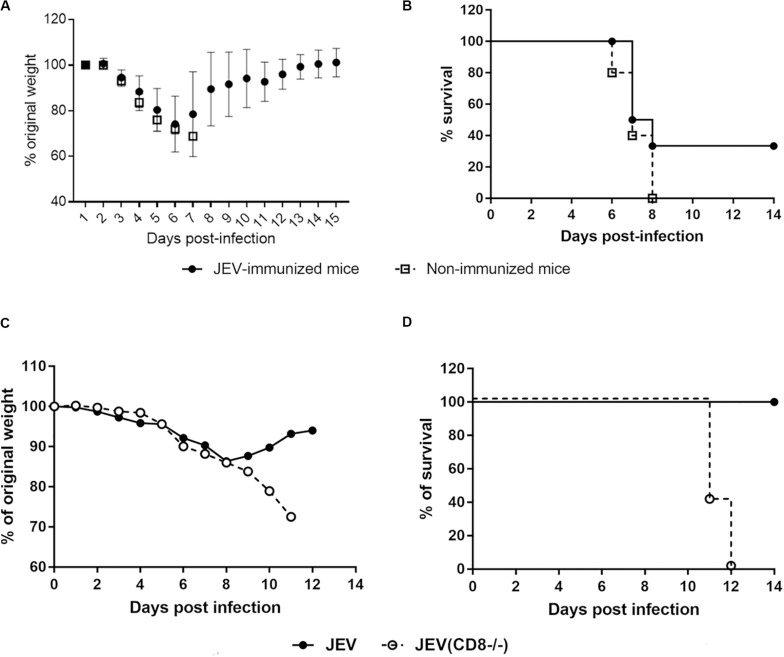
JEV-specific CD8 T cells contribute to the cross-protection against ZIKV in HHD *Ifnar1*^–/–^ mice **(A,B)**. Body weight **(A)** and survival **(B)** of HHD *Ifnar1*^–/–^ mice (*n* = 5 each) that were injected with JEV SA14-14-2 vaccine (*n* = 7) or mock (*n* = 5) two times on days 0 and 28. CD8 T cells were purified and adoptively transferred to naïve mice before ZIKV infection, body weight (A) and survival rate **(B)** were monitored. **(C,D)** CD8 T cells in JEV SA14-14-2-immunized mice were depleted with antibody before ZIKV challenge, body weight **(C)** and survival rate **(D)** of HHD *Ifnar1*^–/–^ mice (*n* = 6 each) were monitored.

To determine the cross-protective role of CD8 T cells against ZIKV infection, we transferred purified CD8 T cells from JEV-immunized mice to naïve HHD *Ifnar1*^–/–^ mice prior to ZIKV infection. After ZIKV challenge, all the control mice lost more than 20% of their initial weight in the first week and had to be sacrificed on day 7 or 8 post infection. Mice that were adoptively transferred with JEV-specific CD8 T cells showed similar weight loss as control mice, but three mice (30%) in this group started to recover from day 6 post infection onward (Figures 5A,B).

To further confirm the protective role of CD8 T cells against ZIKV infection, we performed CD8 T cell depletion study in HHD transgenic mice. As shown in Figures 5C,D, JEV-immunized mice that received isotype control antibody slightly loss weight and recovered rapidly after day 8 post-infection, and all the mice survived. At the opposite, when CD8 T cells were depleted in JEV-immunized group, all the mice in this group continuously lost weight and none of them survived after day 12 post ZIKV infection. Collectively, these data indicate that memory CD8 T cells generated from JEV immunization could provide cross-protection against ZIKV infection in HHD transgenic mice.

## Discussion

In this study, we found abundant cross-reactive epitope candidates that have the potential to be presented by the most prevalent MHC-I molecules in the Asian population. Consistent with previous studies, most of these epitope candidates were mainly located in the E, NS3 and NS5 proteins (21–23). We used HLA-transgenic mice to validate those HLA A2-restricted candidates. Among the candidates, the J12 peptide SLVNGVVKL from JEV appeared to be the most immunogenic and immunodominant epitope. Additionally, JEV vaccination induced memory CD8 T cells that can recognize ZIKV analogs SLINGVVRL and SLVNGVVRL, whereas ZIKV-specific CD8 T cells could also cross-react with J12-specific CD8 T cells. Furthermore, adoptively transfer JEV-specific CD8 T cells also could provide cross-protection against ZIKV infection in HLA-A2 transgenic mice. Taken together, these observations suggest that JEV vaccine immunization induce JEV-specific CD8 T cells, having the potential to provide cross-protection against ZIKV infection in humans.

Previous studies by us and others have independent shown that antibodies against JEV are cross-reactive to ZIKV, but passive transfer of JEV antisera fails to protect ZIKV infection in *Ifnar1*^–/–^ mice (18, 24). Thus, antibody response is unlikely to mediate the protection of JEV vaccine-immunized HLA-transgenic or non-transgenic *Ifnar1*^–/–^ mice from ZIKV infection. Although JEV antibodies exhibit antibody-dependent enhancement (ADE) of infection *in vitro*, the ADE effect was not observed in JEV vaccine-immunized mice or JEV antisera transferred mice (18, 24). It has been shown in Dengue virus infection, efficient CD8 T cells response is sufficient to inhibit ADE in infected mice. Our present study together with previous findings (18, 24) all suggest that cross-reactive JEV-specific CD8 T cells are protective to ZIKV infection in wild-type mice that are not HLA-transgenic (18, 19, 25). Thus, JEV vaccine can be clinically used for the prevention of ZIKV infection and also is at low risk of ADE induction in immune-competent humans.

In this study, we found that JEV vaccine immunization can provide cross-protection, but the adoptive transfer of CD8 T cells from JEV vaccine-immunized mice only partially protected naïve HHD *Ifnar1*^–/–^ mice from ZIKV infection. One possible reason for this partial protection is that adoptive CD8 T cells failed to migrate into ZIKV-infected tissues due to a lack of micromilieu cues. Second, it is also possible that cross-reactive CD4 T cells are also important for cross-protection. Furthermore, JEV SA14-14-2 vaccine, as a live virus, could also activate some innate immune cells upon immunization in mice. Upon activation, innate immune cells become memory-like cells and respond to heterologous pathogens quickly and robustly within a few weeks to months. This process is named as trained immunity (26). Thus, it is also possible that JEV vaccination could induce trained immunity and thereby contribute to partial cross-protection against ZIKV infection.

Besides ZIKV, several flaviviruses, including West Nile virus, Usutu virus, Murray Valley encephalitis virus, Alfuy virus, and Spondweni virus, are also closely related to JEV (27). Whether JEV vaccination could elicit cross-protective immunity against these viruses remain unknown. Furthermore, vaccines for tick-borne encephalitis, yellow fever virus and dengue virus have also been clinically approved in humans (28). It is therefore of interest to study whether immunization of these vaccines in humans could affect other closely related flaviviruses. Understanding which mechanisms are involved in cross-protection against other closely related flavivirus is helpful for developing novel strategies to mitigate or prevent newly emerging flavivirus in future.

## Materials and Methods

### Mice and Ethics Statement

HHD (C57BL/6 background) and WT C57BL/6 mice were originally obtained from Dr. F. Lemonnier (Institute Pasteur Paris) and the Shanghai Laboratory Animal Center (SLAC), respectively. AG6 mice deficient in both IFNAR1 and IFNGR were generated from C57BL/6 background *Ifngr1^–/–^* mice (Jackson laboratories, #003288) and *Ifnar1*^–/–^ A129 mice (B&H company). AG6 mice were backcrossed to C57BL/6 background mice for more than 8 generations. HHD *Ifnar1*^–/–^ mice were further generated by crossing AG6 mice with HHD mice. All mice were maintained and bred at the Institute Pasteur of Shanghai under standard pathogen-free conditions. Animal experiments were carried out in the animal biosafety level 2 laboratory and were approved by the Animal Care and Use Committee of the Institute Pasteur of Shanghai, CAS (Approval number: A2017017).

### Peptide Synthesis

Peptides were synthesized by Genscript with a purity >95% confirmed by HPLC and mass spectrometry. All peptides were dissolved in DMSO at 10 mg/ml and stored at −20°C.

### Viruses, Cells, and Reagents

The African Green kidney cell line Vero, Vero E6 and the Baby Hamster Kidney cell line BHK-21 were grown in Dulbecco’s Modified Eagle Medium (DMEM, Gibco, Suzhou, United States), and human erythroleukemic K562 cells were grown in RPMI 1640 medium (Gibco, Suzhou, United States) at 37°C with 5% CO_2_. All media were supplemented with 10% heat-inactivated fetal bovine serum (FBS, Gibco, Suzhou, United States), 100 U/ml penicillin (Gibco, Suzhou, United States) and 100 μg/ml streptomycin (Gibco, Suzhou, United States). The live-attenuated vaccine strain Japanese encephalitis virus SA14-14-2 was gifted by Dr. Chengfeng Qin, and virus stocks were propagated on BHK-21 cells and titrated on Vero E6 cells by plaque-forming assay. The Zika virus strain SZ-WIV01 (GenBank number KU963796) was obtained from the Microorganisms & Viruses Culture Collection Center, Wuhan Institute of Virology, CAS, and virus stocks were propagated on Vero cells and titrated on Vero E6 cells by plaque-forming assay.

PMA and ionomycin were purchased from Sigma-Aldrich. Brefeldin A was purchased from Biotime. Antibodies against mouse CD8α, CD3, TCRβ, CD44, CD69, IFNγ and TNFα were purchased from eBioscience.

### JEV Vaccine Immunization and Infection With ZIKV

JEV SA-14-14-2 (5 × 10^4^ pfu/mouse) or ZIKV (1 × 10^4^ pfu/mouse) was administered to 6- to 8-week-old HHD mice by intraperitoneal injection (i.p.) or retro-orbital injection. Seven days post infection, splenocytes were collected for flow cytometry analysis or ELISpot assay.

To detect JEV-specific CD8 T cells cross-reactive with ZIKV, 6- to 8-week-old HHD mice were immunized with SA-14-14-2 (i.p., 5 × 10^4^ pfu/mouse) and infected with ZIKV (r.o., 1 × 10^4^ pfu/mouse) 28 days after immunization. Five days post infection, splenocytes were collected for flow cytometry analysis or ELISpot assay.

### Mouse IFNγ ELISpot Assay

Mouse IFNγ ELISpot assay was performed using the ELISpot kit (3321-2A, Mabtech) according to the manufacturer’s instruction. Briefly, the day before the experiment, plates were coated with anti-IFNγ antibody AN18 at 4°C overnight. After the plates were washed with PBS and blocked with 10% FBS in RPMI, 0.4 μg of each peptide and 2.5 × 10^5^ cells/well were added. The plates were incubated for 40 hours and then washed and visualized. Wells containing splenocytes in the presence of anti-CD3 and anti-CD28 antibodies were used as positive controls, while negative controls contained splenocytes in the presence of DMSO.

### ZIKV Challenge in HHD *Ifnar1*^–/–^ Mice

Four-week-old HHD *Ifnar1*^–/–^ mice were immunized with two doses of 5 × 10^4^ pfu JEV SA14-14-2 four weeks apart. Two weeks after the second immunization, the mice were infected with 10^4^ pfu of ZIKV SZ-WIV01. Mouse survival, weight change and clinical score were monitored over 2 weeks. Clinical scores were graded as follows: 0 = healthy; 1 = ruffled fur; 2 = hunched position or reduced activity; 3 = limb weakness; 4 = paralyzed and 5 = moribund or dead.

For CD8 T cell adoptive transfer, 6- to 8-week-old HHD mice were immunized with 5 × 10^4^ pfu of JEV SA14-14-2. Four weeks post immunization, splenocytes were collected, and CD8 T cells were isolated using the EasySep^TM^ Mouse CD8 + T Cell Isolation Kit according to the manufacturer’s specifications. Then, 5-week-old HHD *Ifnar1*^–/–^ mice were injected with 7.5 million purified CD8-positive splenocytes *via* the i.p. route. One day later, recipient mice were inoculated with 2 × 10^5^ pfu ZIKV, and their survival and weight change were assessed for 2 weeks.

### Flow Cytometry

Cells were washed and blocked in staining buffer (PBS, 0.3% BSA and 0.1% sodium azide) containing the anti-CD16/CD32 antibody for 10 min at 4°C and then stained with fluorophore-conjugated antibodies. After the cells were washed twice with staining buffer, data were collected on a Fortessa flow cytometer (BD Biosciences). For intracellular staining, 2.5 μg/ml BFA was added during the last 4 h of stimulation to block the secretion of cytokines. After the cells were washed and stained for cell-surface markers and fixated and permeabilized with the IC fixation buffer Kit (eBioscience) according to the manufacturer’s protocol, the cells were stained with FITC-anti-mouse IFNγ or isotype control and analyzed with a Fortessa flow cytometer. The data were analyzed using FlowJo software.

### Statistical Analyses

Statistical analyses for continuous data were performed with Prism6 for Windows software (Prism Graph-Pad Software Inc.). *P* < 0.05 was considered significant. Graphs were produced, and statistical analyses were performed using GraphPad Prism.

## Data Availability Statement

All datasets presented in this study are included in the article/Supplementary Material.

## Ethics Statement

The animal study was reviewed and approved by Shanghai Laboratory Animal Center.

## Author Contributions

QL and XL designed this research. MT, WD, GH, YX, JS, SG, and XC performed the research. CQ, LL, and JZ contributed to reagents and animals. MT, WD, LL, and SG analyzed the data. MT, WD, and QL wrote the manuscript. All authors contributed to the article and approved the submitted version.

## Conflict of Interest

The authors declare that the research was conducted in the absence of any commercial or financial relationships that could be construed as a potential conflict of interest.
