# Supplementary material for: Japanese Encephalitis Virus Vaccination Elicits Cross-Reactive HLA-Class I-Restricted CD8 T Cell Response Against Zika Virus Infection
Source: Front Immunol. 2020 Sep 25;11:577546. doi: 10.3389/fimmu.2020.577546 (PMC7546338; doi:10.3389/fimmu.2020.577546)
Supplement: Supplementary file 1 [file Data_Sheet_1.PDF]

Supplementary Table 1. IEDB prediction of ZIKV and JEV HLA-B*58:01-restricted epitope presenting sequence homologies

Polyprotein domain	Virus strain	Start	End	Sequence	IEDB score
E	ZIKV MR766	392	400	GNGCGLFGK	2.8
	JEV SA14	396	404	GNGCGFFGK	2.1
E	ZIKV MR766	400	408	KGSLVTCAK	1.45
	JEV SA14	404	412	KGSIDTCAK	2.75
E	ZIKV MR766	618	626	YAGTDGPCK	3.2
	JEV SA14	622	630	YSGSDGPCK	2.35
E	ZIKV MR766	672	680	YIVIGVGDK	2.75
	JEV SA14	676	684	YIVVGRGDK	2.9
NS2A	ZIKV MR766	1218	1226	HLALVAAFK	0.9
	JEV SA14	1222	1230	HLALIAVFK	0.85
NS2A	ZIKV MR766	1407	1415	IVSYVVSGK	0.45
	JEV SA14	1413	1421	AVSYVVSGK	0.35
NS3	ZIKV MR766	1537	1545	GVMQEGVFH	2
	JEV SA14	1543	1551	GVMYENVFH	1.45
NS3	ZIKV MR766	1660	1668	VSAITQGKR	3.3
	JEV SA14	1666	1674	VSAIVQGDR	3.7
NS3	ZIKV MR766	1716	1724	RTVILAPTR	2.25
	JEV SA14	1722	1730	RTAVLAPTR	1.6
NS3	ZIKV MR766	1857	1865	TVWFVPSVR	1.1
	JEV SA14	1863	1871	TVWFVASVK	0.35
NS3	ZIKV MR766	1946	1954	VTHASAAQR	1.5
	JEV SA14	1954	1962	ITSASAAQR	1.3
NS3	ZIKV MR766	1949	1957	ASAAQRRGR	3.05
	JEV SA14	1957	1965	ASAAQRRGR	3.05
NS4A	ZIKV MR766	2232	2240	VVLIPEPEK	0.9
	JEV SA14	2239	2247	VVLIPEPEK	0.9
NS5	ZIKV MR766	2592	2600	KVVDLGCGR	2.05
	JEV SA14	2603	2611	KVIDLGCGR	1.3
NS5	ZIKV MR766	2604	2612	SYYAATIRK	0.45
	JEV SA14	2615	2623	SYYAATLKK	0.45
NS5	ZIKV MR766	2613	2621	VQEVRGYTK	2.2
	JEV SA14	2624	2632	VQEVRGYTK	2.2
NS5	ZIKV MR766	2635	2643	YGWNIVRLK	2.95
	JEV SA14	2646	2654	YGWNLVSLK	1.45
NS5	ZIKV MR766	2721	2729	GLVRVPLCR	3.6
	JEV SA14	2732	2740	GLVRLPLSR	3

NS5	ZIKV MR766	2751	2759	TTSQLLLGR	0.8
	JEV SA14	2762	2770	MTSQVLLGR	0.8
NS5	ZIKV MR766	2818	2826	RTWAYHGSY	1.4
	JEV SA14	2830	2838	RTWTYHGSY	1.55
NS5	ZIKV MR766	2835	2843	SSLVNGVVR	1.7
	JEV SA14	2847	2855	SSLVNGVVK	0.55
NS5	ZIKV MR766	2867	2875	GQQRVFKEK	1.4
	JEV SA14	2879	2887	GQQRVFKEK	1.4
NS5	ZIKV MR766	2911	2919	CTKEEFINK	0.6
	JEV SA14	2923	2931	CTKEEFIKK	0.75
NS5	ZIKV MR766	2966	2974	SCVYNMMGK	3.55
	JEV SA14	2978	2986	TCIYNMMGK	2.85
NS5	ZIKV MR766	2967	2975	CVYNMMGKR	1.2
	JEV SA14	2979	2987	CIYNMMGKR	1.5
NS5	ZIKV MR766	2970	2978	NMMGKREKK	1.2
	JEV SA14	2982	2990	NMMGKREKK	1.2
NS5	ZIKV MR766	3105	3113	KTVMDIISR	1.2
	JEV SA14	3117	3125	KTVMDVISR	1.15
NS5	ZIKV MR766	3178	3186	VSGDDCVVK	1.05
	JEV SA14	3192	3200	ISGDDCAVK	1.6
NS5	ZIKV MR766	3276	3284	YAQMWQLLY	2.5
	JEV SA14	3290	3298	YAQMWVLLY	2.05
NS5	ZIKV MR766	3277	3285	AQMWQLLYF	2.85
	JEV SA14	3291	3299	AQMWVLLYF	2.05
NS5	ZIKV MR766	3278	3286	QMWQLLYFH	3.5
	JEV SA14	3292	3300	QMWVLLYFH	3.3
NS5	ZIKV MR766	3310	3318	RTTWSIHGK	0.45
	JEV SA14	3324	3332	RTSWSIHSK	0.2
NS5	ZIKV MR766	3351	3359	WTDIPYLGK	1.1
	JEV SA14	3365	3373	WTDVPYVGK	1.45
NS5	ZIKV MR766	3366	3374	GSLIGHRPR	3.4
	JEV SA14	3380	3388	GSLIGTRSR	3.9

Supplementary Table 2. IEDB prediction of ZIKV and JEV HLA-A*11:01-restricted epitope presenting sequence homologies

Polyprotein domain	Virus strain	Start	End	Sequences	IEDB score
E	ZIKV MR766	392	400	GNGCGLFGK	2.8
	JEV SA14	396	404	GNGCGFFGK	2.1
E	ZIKV MR766	400	408	KGSLVTCAK	1.45
	JEV SA14	404	412	KGSIDTCAK	2.75
E	ZIKV MR766	618	626	YAGTDGPCK	3.2
	JEV SA14	622	630	YSGSDGPCK	2.35
E	ZIKV MR766	672	680	YIVIGVGDK	2.75
	JEV SA14	676	684	YIVVGRGDK	2.9
NS2A	ZIKV MR766	1218	1226	HLALVAAFK	0.9
	JEV SA14	1222	1230	HLALIAVFK	0.85
NS2A	ZIKV MR766	1407	1415	IVSYVVSGK	0.45
	JEV SA14	1413	1421	AVSYVVSGK	0.35
NS3	ZIKV MR766	1537	1545	GVMQEGVFH	2
	JEV SA14	1543	1551	GVMYENVFH	1.45
NS3	ZIKV MR766	1660	1668	VSAITQGKR	3.3
	JEV SA14	1666	1674	VSAIVQGDR	3.7
NS3	ZIKV MR766	1716	1724	RTVILAPTR	2.25
	JEV SA14	1722	1730	RTAVLAPTR	1.6
NS3	ZIKV MR766	1857	1865	TVWFVPSVR	1.1
	JEV SA14	1863	1871	TVWFVASVK	0.35
NS3	ZIKV MR766	1946	1954	VTHASAAQR	1.5
	JEV SA14	1954	1962	ITSASAAQR	1.3
NS3	ZIKV MR766	1949	1957	ASAAQRRGR	3.05
	JEV SA14	1957	1965	ASAAQRRGR	3.05
NS4A	ZIKV MR766	2232	2240	VVLIPEPEK	0.9
	JEV SA14	2239	2247	VVLIPEPEK	0.9
NS5	ZIKV MR766	2592	2600	KVVDLGCGR	2.05
	JEV SA14	2603	2611	KVIDLGCGR	1.3
NS5	ZIKV MR766	2604	2612	SYYAATIRK	0.45
	JEV SA14	2615	2623	SYYAATLKK	0.45
NS5	ZIKV MR766	2613	2621	VQEVRGYTK	2.2
	JEV SA14	2624	2632	VQEVRGYTK	2.2
NS5	ZIKV MR766	2635	2643	YGWNIVRLK	2.95
	JEV SA14	2646	2654	YGWNLVSLK	1.45
NS5	ZIKV MR766	2721	2729	GLVRVPLCR	3.6
	JEV SA14	2732	2740	GLVRLPLSR	3

NS5	ZIKV MR766	2751	2759	TTSQLLLGR	0.8
	JEV SA14	2762	2770	MTSQVLLGR	0.8
NS5	ZIKV MR766	2818	2826	RTWAYHGSY	1.4
	JEV SA14	2830	2838	RTWTYHGSY	1.55
NS5	ZIKV MR766	2835	2843	SSLVNGVVR	1.7
	JEV SA14	2847	2855	SSLVNGVVK	0.55
NS5	ZIKV MR766	2867	2875	GQQRVFKEK	1.4
	JEV SA14	2879	2887	GQQRVFKEK	1.4
NS5	ZIKV MR766	2911	2919	CTKEEFINK	0.6
	JEV SA14	2923	2931	CTKEEFIKK	0.75
NS5	ZIKV MR766	2966	2974	SCVYNMMGK	3.55
	JEV SA14	2978	2986	TCIYNMMGK	2.85
NS5	ZIKV MR766	2967	2975	CVYNMMGKR	1.2
	JEV SA14	2979	2987	CIYNMMGKR	1.5
NS5	ZIKV MR766	2970	2978	NMMGKREKK	1.2
	JEV SA14	2982	2990	NMMGKREKK	1.2
NS5	ZIKV MR766	3105	3113	KTVMDIISR	1.2
	JEV SA14	3117	3125	KTVMDVISR	1.15
NS5	ZIKV MR766	3178	3186	VSGDDCVVK	1.05
	JEV SA14	3192	3200	ISGDDCAVK	1.6
NS5	ZIKV MR766	3276	3284	YAQMWQLLY	2.5
	JEV SA14	3290	3298	YAQMWVLLY	2.05
NS5	ZIKV MR766	3277	3285	AQMWQLLYF	2.85
	JEV SA14	3291	3299	AQMWVLLYF	2.05
NS5	ZIKV MR766	3278	3286	QMWQLLYFH	3.5
	JEV SA14	3292	3300	QMWVLLYFH	3.3
NS5	ZIKV MR766	3310	3318	RTTWSIHGK	0.45
	JEV SA14	3324	3332	RTSWSIHSK	0.2
NS5	ZIKV MR766	3351	3359	WTDIPYLGK	1.1
	JEV SA14	3365	3373	WTDVPYVGK	1.45
NS5	ZIKV MR766	3366	3374	GSLIGHRPR	3.4
	JEV SA14	3380	3388	GSLIGTRSR	3.9

Supplementary Table 3. IEDB prediction of ZIKV and JEV HLA-A*24:02-restricted epitope presenting sequence homologies

Polyprotein domain	Virus strain	Start	End	Sequences	IEDB score
M	ZIKV MTR766	283	291	LLIAPAYSI	3.75
	JEV SA14	287	295	LLVAPAYSF	2.9
E	ZIKV MTR766	390	398	GWGNGCGLF	1.1
	JEV SA14	394	402	GWGNGCGFF	1.35
E	ZIKV MTR766	503	511	WFHDIPLPW	1.2
	JEV SA14	511	519	WFHDLALPW	2.15
E	ZIKV MTR766	716	724	DFGSVGGVF	2.15
	JEV SA14	720	728	DFGSIGGVF	2.15
NS1	ZIKV MTR766	985	992	VHSDLGYWI	3.7
	JEV SA14	988	996	VHSDLSYWI	2.75
NS3	ZIKV MTR766	1658	1666	SYVSAITQG	3.9
	JEV SA14	1664	1672	SYVSAIVQG	3.05
NS3	ZIKV MTR766	1811	1819	IFMTATPPG	3.55
	JEV SA14	1817	1825	IFMTATPPG	3.55
NS3	ZIKV MTR766	1823	1831	AFPDSNSPI	2.2
	JEV SA14	1829	1837	PFPDSNAPI	2.25
NS3	ZIKV MTR766	2044	2052	AYQVASAGI	0.65
	JEV SA14	2052	2060	AYKVASNGI	0.85
NS3	ZIKV MTR766	2053	2061	TYTDRRWCF	0.35
	JEV SA14	2061	2069	QYTDRKWCF	0.6
NS4B	ZIKV MTR766	2464	2472	ITAATSTLW	1.45
	JEV SA14	2474	2482	VTAATLTLW	2.35
NS5	ZIKV MTR766	2604	2612	SYYAATIRK	3.7
	JEV SA14	2615	2623	SYYAATLKK	2.2
NS5	ZIKV MTR766	2605	2613	YYAATIRKV	1
	JEV SA14	2616	2624	YYAATLKKV	1.2
NS5	ZIKV MTR766	2629	2637	PMLVQSYGW	3.4
	JEV SA14	2640	2648	PMLMQSYGW	3.35
NS5	ZIKV MTR766	2631	2638	LVQSYGWNI	2.8
	JEV SA14	2642	2650	LMQSYGWNL	2.75
NS5	ZIKV MTR766	2634	2642	SYGWNIVRL	0.65
	JEV SA14	2645	2653	SYGWNLVSL	0.85
NS5	ZIKV MTR766	2768	2776	KYEEDVNLG	3.3
	JEV SA14	2780	2788	KYEEDVNLG	3.3
NS5	ZIKV MTR766	2922	2930	SNAALGAIF	3.7
	JEV SA14	2934	2942	SNAALGAVF	3.9
NS5	ZIKV MTR766	2987	2995	GSRAIWYMW	4.1
	JEV SA14	2999	3007	GSRAIWFMW	2.75
NS5	ZIKV MTR766	2995	3003	WLGARFLEF	1.8

	JEV SA14	3007	3015	WLGARYLEF	1.35
NS5	ZIKV MTR766	2999	3007	RFLEFEALG	5
	JEV SA14	3011	3019	RYLEFEALG	1.15
NS5	ZIKV MTR766	3047	3055	MYADDTAGW	0.65
	JEV SA14	3059	3067	MYADDTAGW	0.65
NS5	ZIKV MTR766	3089	3097	TYQNKVVKV	1.6
	JEV SA14	3101	3109	TYRHKVVKV	3
NS5	ZIKV MTR766	3122	3130	VVTYALNTF	2.2
	JEV SA14	3134	3142	VVTYALNTF	2.2
NS5	ZIKV MTR766	3124	3132	TYALNTFTN	0.65
	JEV SA14	3136	3144	TYALNTFTN	0.65
NS5	ZIKV MTR766	3191	3199	RFAHALRFL	1.5
	JEV SA14	3205	3213	RFATALHFL	0.75
NS5	ZIKV MTR766	3223	3231	EVPFCSHHF	2.95
	JEV SA14	3237	3245	QVPFCSNHF	3.05
NS5	ZIKV MTR766	3276	3283	SYAQMWQLL	0.15
	JEV SA14	3289	3297	AYAQMWVLL	0.3
NS5	ZIKV MTR766	3277	3285	AQMWQLLYF	1.05
	JEV SA14	3291	3299	AQMWVLLYF	0.9
NS5	ZIKV MTR766	3279	3287	MWQLLYFHR	1.65
	JEV SA14	3293	3301	MWVLLYFHR	2
NS5	ZIKV MTR766	3284	3292	YFHRRDLRL	2.7
	JEV SA14	3298	3306	YFHRRDLRL	2.7
NS5	ZIKV MTR766	3297	3305	ICSAVPVDW	3.2
	JEV SA14	3311	3319	ICSAVPVDW	3.2
NS5	ZIKV MTR766	3307	3315	PTGRTTWSI	3.45
	JEV SA14	3321	3329	PTGRTSWSI	3.55
NS5	ZIKV MTR766	3308	3316	PYRTWAYHG	5
	JEV SA14	2828	2836	PYRTWTYHG	4
NS5	ZIKV MTR766	3355	3363	PYLGKREDL	2.95
	JEV SA14	3369	3377	PYVGKREDI	1.95
